# Biodegradable Carriers for Delivery of VEGF Plasmid DNA for the Treatment of Critical Limb Ischemia

**DOI:** 10.3389/fphar.2017.00528

**Published:** 2017-08-09

**Authors:** Guang Liu, Zhiwei Fang, Minglu Yuan, Weimin Li, Yunqi Yang, Mier Jiang, Yuanming Ouyang, Weien Yuan

**Affiliations:** ^1^Department of Vascular Surgery, Shanghai Ninth People's Hospital, Shanghai Jiao Tong University School of Medicine Shanghai, China; ^2^School of Pharmacy, Shanghai Jiao Tong University Shanghai, China; ^3^Shanghai Sixth People's Hospital, Shanghai University of Medicine and Health Shanghai, China

**Keywords:** polyethylenimine, 2,6-pyridinedicarboxaldehyde, vascular endothelial factor, peripheral ischemic artery disease

## Abstract

The safe and efficient delivery of therapeutic nucleic acid is a prerequisite for an effective DNA therapy. In this study, we condensed the low molecular weight polyethylenimine (PEI, 1.8k Da) with 2,6-pyridinedicarboxaldehyde (PDA), both of which are degradable *in vivo*, to synthesize a biodegradable polycationic material (PDAPEI) to deliver vascular endothelial growth factor (VEGF) plasmid DNA (pDNA). Particle size and zeta potential of this novel degradable PEI derivatives-pDNA nanoparticle were investigated and *in vitro* cytotoxicity was estimated on human umbilical vein endothelial cells (HUVECs). Using pDNA-encoding VEGF-A and green fluorescence protein (GFP), we also checked transfection efficiency of the vector (PDAPEI) and found its excellent performance at 40 w/w ratio. We successfully established peripheral ischemia animal model on C57/BL6J mice to evaluate the therapeutic effect of PDAPEI/pVEGF-A polyplex system on ischemic disease and a conclusion was made that PDAPEI is a promising gene vector in the treatment of peripheral ischemic artery disease (PAD).

## Introduction

Peripheral ischemic artery disease (PAD) is a congenital or acquired vascular disease caused by inadequate blood supply to a tissue or organ, in which the most severe form is critical limb ischemia (CLI; Shishehbor et al., 2016; Rundback et al., 2017). As the clinical end stage of PAD, CLI has a negative prognosis and is often associated with significant morbidity, mortality, and amputation rates (Varu et al., 2010; Farber and Eberhardt, 2016; Schreve et al., 2017). Current pharmacological treatments include revascularization, therapeutic angiogenesis, hyperbaric oxygen, and venous arterialization (Li et al., 2015; Farber and Eberhardt, 2016; Schreve et al., 2017). Therapeutic angiogenesis with proangiogenic proteins, genes, and stem cells has been tested pre-clinically to stimulate the growth of blood vessels (Bauters et al., 1995; Sun et al., 2005; Jazwa et al., 2015; Sanada et al., 2015; Beegle et al., 2016). Vascular endothelial growth factor (VEGF) is one of the signal proteins that could stimulate vasculogenesis and angiogenesis (Nissen et al., 1998; Bobek et al., 2006; Ganta et al., 2017). VEGF family comprises five members in mammals, including VEGF-A, B, C, D, and placenta growth factor (Takahashi and Shibuya, 2005). When a cell is deficient in oxygen, hypoxia-inducible factor (HIF) would be induced, and then HIF stimulates the release of VEGF-A, which could relieve the situation of endothelial cell death and vascular regression (Carmeliet, 2000; Smith et al., 2008).

Despite the evidence that ischemic tissues are responsible for the stimulation of VEGF-A translated by the plasmid DNA (pDNA), inefficiency in direct gene delivery to target cells has been a barrier to a promising therapy. To overcome this problem, viral vectors or non-viral vectors for gene delivery are designed (Lungwitz et al., 2005; Rogers et al., 2014; Boden et al., 2016; Tan et al., 2016). With relatively high transfection efficiency, viral vectors could successfully deliver genes into cells, but several adverse effects such as immunogenicity and oncogenicity limit its application principally (Thomas et al., 2003; Mancheno-Corvo and Martin-Duque, 2006; Stewart et al., 2016). On the contrary, non-viral vectors are often preferred for its low toxicity and amenability to both *in vivo* and *in vitro* translation, but its low transfection efficiency sometimes hindered the application in clinical and pre-clinical research (Mintzer and Simanek, 2009; Stewart et al., 2016). Thus, the ideal transport vector should possess the characteristics of both low toxicity and high transfection efficiency simultaneously. Polycationic carrier is often thought to be a good choice among all available non-viral vectors (Chen et al., 2013, 2014, 2016a,b; Ma et al., 2013; Ge et al., 2014a,b; Islam et al., 2014). Characterized by high positive charge and enhanced “proton sponge effect” in endolysosome, polyethylenimine (PEI) is one of the most effective non-viral vectors in gene delivery system (Nel et al., 2009; Neuberg and Kichler, 2014; Xia et al., 2015; Cooper and Putnam, 2016). However, accumulating evidence showed that both transfection efficiency and toxicity of PEI correlate with the molecular weight (Fischer et al., 1999; Guo et al., 2017). PEI with lower molecular weight (such as, 1.8k Da) is generally less toxic, but less efficient. Inspired by the existing strategies to improve the transfection efficiency and lower toxicity at the same time (Duan S. et al., 2012), our laboratory synthesized a new gene carrier formed by linking PEI (1.8k Da) and 2,6-pyridinedicarboxaldehyde (PDA) through bisimine bonds, which were thought to be liable in the acid environment (Kim et al., 2005). The new polymer was named as PDAPEI (Che et al., 2016; Song et al., 2016, 2017).

In this study, we investigated particle size and zeta potential of novel biodegradable polyethylenimine derivatives-pDNA nanoparticles, and estimated cytotoxicity on human umbilical vein endothelial cells (HUVECs) by Cell Counting Kit-8 (CCK-8). Using pDNA encoding VEGF-A and GFP, we also checked transfection efficiency of the new polymers. We successfully established peripheral ischemia animal model on C57/BL6J mice to evaluate the therapeutic effect of PDAPEI/pDNA polyplex system on ischemic disease with plasmid with VEGF-A sequence.

## Materials and methods

### Materials

Branched PEI (molecular weight 1.8k and 25k Da) and anhydrous ethylene dichloride (EDC) were purchased from Sigma-Aldrich. 2,6-pyridinedicarboxaldehyde (PDA) was obtained from TCI (Shanghai) Development Co., Ltd. Cellulose membranes (MWCO = 10,000 Da), Roswell Park Memorial Institute-1640 (RPMI-1640) medium, Fetal Bovine Serum (FBS), and Phosphate Buffered Saline (PBS, pH 7.4 basic) were purchased from Thermo Fisher Scientific (Shanghai). *Escherichia coli* bacterial strain DH5a was obtained from Tiangen Biotech (Beijing) CO., Ltd. The plasmids pVEGF165 and pGFP were constructed previously in our laboratory. Water was purified using a milli-Q instrument (Millipore).

### Methods

#### PDA-PEI conjugation

The synthesis of PDA-PEI polymer was carried out as previously reported (Che et al., 2016; Song et al., 2016). Initially, 1 mmol PEI (1.8k Da) was dissolved in 20 mL anhydrous EDC under vigorous magnetic stirring. 2 mmol PDA dissolved in 20 ml anhydrous EDC was introduced into PEI solutions dropwise with vigorous stirring. The reaction lasted for 48 h at room temperature. After the removal of organic solvent through evaporation, the terminal product was dialyzed through the cellulose membranes (MWCO = 10,000 Da). The final yellow polymer PDAPEI was obtained after lyophilization of 2 days.

#### Characterization of PDAPEI

The structure and average molecular weight (M_w_) of PDAPEI were confirmed by Fourier Transform Infrared spectrometry (FTIR), ^1^H-Nuclear Magnetic Resonance (^1^H-NMR; Che et al., 2016; Song et al., 2016), and Gel Permeation Chromatography (GPC). ^1^H-NMR spectrum was obtained in DMSO-d_6_ with 0.03% (v/v) tetramethylsilane (TMS) as internal standard using a Varian Mercury Plus 400 MHz spectrometer. FTIR spectrum was recorded in a KBr pellet using a Bruker Optics FTIR spectrometer. In GPC test, the M_w_ and polydispersity index (PDI) were obtained by an Agilent 1260 Infinity with a series of polyethylene glycol (PEG) standards and 25k Da PEI for calibration. The system was equipped with a diode array detector (DAD) and refractive index detector (RID) with two columns in a guard column and a PL aquagel-OH column at 40°C. As an eluent, 0.05% NaN_3_ at a flow rate of 1.0 mL/min was used.

#### Preparation and purification of pDNA

In the present study, the plasmid was amplified in *Escherichia coli* bacterial strain DH5a and then extracted from overnight bacterial cultures using EndoFreeTM Plasmid Maxi (Qiagen) according to the manufacturer's instructions. The purity of the product was determined by measuring the ratio of its absorbance at 260 and 280 nm (A260/A280) using UV spectrophotometry. A260/A280 value of the pDNA we used in this study was between 1.8 and 2.0, which confirmed the purity.

#### Preparation of PDAPEI/pDNA and PEI (2.5k Da)/pDNA polyplexes

PDAPEI/pDNA polyplexes were prepared at a range of carrier to pDNA mass ratios (weight/weight, w/w). First, pDNA solution (20 μg/ml) and PDAPEI stock solution (2 mg/ml) were obtained by dilution and dissolution, respectively, with deionized water. Then, different concentrations of PDAPEI solutions were obtained in gradient by further dilution of this stock solution. These PDAPEI solutions with various concentrations were, respectively, added into pDNA solution to prepare polyplexes. The final concentration of pDNA in all samples was 10 μg/ml. These samples were incubated for 30 min at room temperature. The w/w ratios of PDAPEI/pDNA polyplexes we chose in this study were 10, 20, 30, 40, and 50. Similarly, PEI (2.5k Da)/pDNA polyplex at 2 w/w ratio (optimal mass ratio), which is currently the golden standard for *in vitro* transfection (Boussif et al., 1995; Wang et al., 2011; Duan S. Y. et al., 2012; Xiang et al., 2012a; Che et al., 2016), was prepared as a control group.

#### Characterization of prepared polyplexes

##### Agarose gel electrophoresis

PDAPEI/pDNA polyplexes at different ratios (10, 20, 30, 40 and 50; 0.05, 0.1, 0.2, 0.3, 0.4, 0.5) were loaded on 1.0% (w/v) agarose gel in Tris-Acetate-EDTA (TAE) running buffer for 45 min at 130 V. Image of Agarose gel with DNA bands was visualized in Tanon-3500 Gel Imaging System. Naked pDNA was set as a control group.

##### Particle size, zeta potential and morphology measurements

Particle-Size Analyzer (Brookhaven Instruments) was utilized to measure the particle size and PDI of PDAPEI/pDNA polyplexes at various ratios of w/w, whereas zeta potential was measured by Zeta Potential Analyzer (Zetasizer Nano, Malvern Instruments). All these measurements were under the temperature of 37°C. The morphology of PDAPEI/pDNA polyplex at 40 w/w ratio was observed by Transmission Electron Microscope (TEM, JEOL JEM 2010 system).

#### *In vitro* cell transfection

In this study, the pDNA-encoding GFP was used as an alternative to evaluate the transfection efficiency of PDAPEI/ pDNA polyplexes. HUVECs (5–10 × 10^4^ cells/ml) were seeded in 48-well plates with 500 μl RPMI-1640 medium containing 10% FBS and 1% antibiotics at 37°C in a 5% CO_2_ humidified atmosphere. When each well was confluent around 90%, 50 μl PDAPEI/pDNA polyplexes solutions at various w/w ratios (10, 20, 30, 40, and 50), PEI (2.5k Da)/pDNA polyplexes solution (positive control), and naked pDNA solution (10 μg/ml, negative control) were transfected into the cells for 4 h in the fresh 250 μl RPMI-1640 medium. Afterwards, the medium was replaced by 500 μl RPMI-1640 medium containing 10% FBS and then further incubated for 48 h. HUVECs were then washed with PBS solution and detached with 0.25% trypsin-EDTA solution, followed by centrifugation at 1500 rpm for 5 min (Eppendorf 5810 R Centrifuge, Germany). The supernatant fluid was replaced by 500 μl PBS buffer, followed by pipetting to mix evenly. To identify the expression level of GFP, the percentage of transfected positively cells was quantified by flow cytometer (BD FACSCalibur).

#### *In vitro* cytotoxicity

The cytotoxicity effects of PDAPEI/pDNA and PEI/pDNA polyplexes against HUVECs were evaluated by CCK-8 reagent. HUVECs (1 × 10^4^ cells/ml) were cultured in 96-well plates with 500 μl RPMI-1640 medium containing 10% FBS and 1% antibiotics at 37°C in a 5% CO_2_ humidified atmosphere for 24 h. 10 μl PEI (2.5k Da)/pDNA polyplexes solution (positive control) and PDAPEI/pDNA polyplexes solutions at various weight ratios (10, 20, 30, 40, and 50) were transfected into the cells for 4 h in the fresh 250 μl RPMI-1640 medium. Afterwards, 10 μl CCK-8 reagent was added into each well and further incubated for 2 h. Multifunctional microplate reader (SpectraMax M3 Multi-Mode Microplate Reader) was utilized to measure the absorbance of each well at 450 and 630 nm. RPMI-1640 medium alone was considered as a blank control group, whereas HUVECs not transfected by polyplexes was the negative control group. Cell viability was calculated by the following formula.

$$\begin{array}{l} \text{Cell}\;\text{viability}\;\left(\%\right)=\frac{\text{OD}\;\left(\text{Sample}\right)-\text{OD}\;\left(\text{Blank}\right)}{\text{OD}\;\left(\text{Negative}\right)-\text{OD}\;\left(\text{Blank}\right)}*100\%\\ \text{ OD}={\text{OD}}_{450\text{nm}}-{\text{OD}}_{630\text{nm}} \end{array}$$

#### *In vivo* treatment in peripheral ischemia animal model

Peripheral ischemia animal model was established on C57/BL6J mice through the way of hind limb ischemia surgery to evaluate the therapeutic effect of PDAPEI/pDNA polyplex system. Briefly, the C57/BL6J female mice (4 weeks, weight of 20 ± 2 g) were maintained in the SPF environment for 1 week and then anesthetized by intraperitoneal injection of 5% urethane solution (5 ml/kg). Afterwards, an incision of the skin was made from the medial thigh to the knee and the membranes covering the muscle were dissected away. The neurovascular bundle was exposed by piercing the membranous femoral sheath, and the external iliac artery, femoral artery, and peroneal artery were separated, respectively. Ligations were performed in proximal and distal femoral artery with 5/0 polypropylene suture (Premilene, Braun, Melsungen AG) and a cut out was made between two ligations (Sun et al., 2005; Chen et al., 2007; Niiyama et al., 2009). Then, animals were sutured and received intraperitoneal injection of 10% ampicillin (10 ml/kg). Before they revived, 0.2 ml 5% glucose solution/4 h was continuingly given to C57/BL6J mice by oral gavage. The treated C57/BL6J female mice were divided randomly into 3 groups (A, B, and C, 20 mice per group). Each group (A, B, and C) was further divided into 4 subgroups: Saline (blank, *n* = 5), naked pDNA (negative, *n* = 5), PEI (25k Da)/pDNA (positive, *n* = 5), and PDAPEI/pDNA (*n* = 5). For treatment groups, 0.1 ml solutions containing 10 μg pDNA (freshly prepared) were injected to the mice once every 2 days. The growth situation of hind limb was monitored for 28 days.

#### Expression level examination *in vivo*

To identify the transfection efficiency of polyplex in cells and detect the VEGF-A protein expression level *in vivo*, we measured the concentration of expressed VEGF-A protein in hind limb homogenate at Day 7 (group A) and Day 14 (group B) by using Mouse VEGF-A ELISA Kit according to the manufacturer's instructions.

#### Immuno-histological examination and microvessel quantification

To get a clear detection of the cell propagation in hind limb, mice of group C were further injected intramuscularly with BrdU solution (30 mg/Kg) at Day 2 and raised for 28 days. Double immunofluorescent staining for endothelial cell marker CD31 (Schlossman, 1995) and BrdU was made to observe the microvessel density in peripheral ischemic muscle tissues of group C mice when they were sacrificed by cervical dislocation at Day 28. The vessel densities calculated from the slides were demonstrated as microvessel per millimeter squared. In this study, ImageJ software was utilized to determine manually the number of positively stained blood vessels, while the staining consisting of a single cell was not counted as a capillary (Chen et al., 2007).

#### Ethics statement

This study was carried out in accordance with the recommendations of the National Institutes of Health Guide for care and Use of Laboratory Animals, China (GB14925-2010) and the Regulations for the Administration of Affairs Concerning Experimental Animals (China, 2014). The protocol was approved by the Committee for the Administration of Affair Concerning Laboratory Animals for Shanghai Jiao Tong University.

#### Statistical analysis

The data of the results were expressed as mean ± standard deviation (*SD*). The analysis of variance (ANOVA) with Tukey's multiple comparison test was performed between means with SPSS 22.0 software and a value for ^*^P < 0.05, ^**^P < 0.01, and ^***^P < 0.001 was considered significant statistically.

## Results

### Characterization of PDAPEI

**Figure 1:**
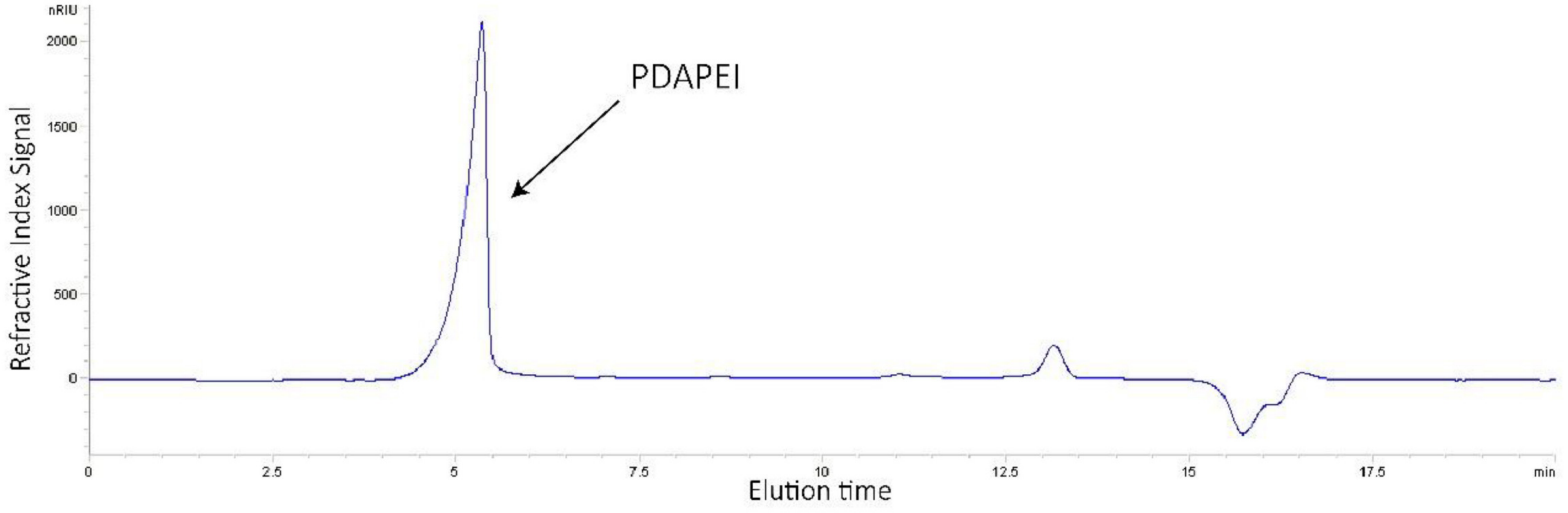
GPC spectrum of synthetic polymer PDAPEI.

The structure of synthetic polymer PDAPEI was confirmed by IR and ^1^H NMR (Che et al., 2016). In the GPC test (Figure 1), M_w_ of polymer PDAPEI was determined to be 11.8k Da, and the PDI was 1.76. All these results from NMR, IR, and GPC confirmed the successful formation of PDAPEI.

### Characterization of PDAPEI/pDNA polyplex

#### Agarose gel electrophoresis

**Figure 2:**
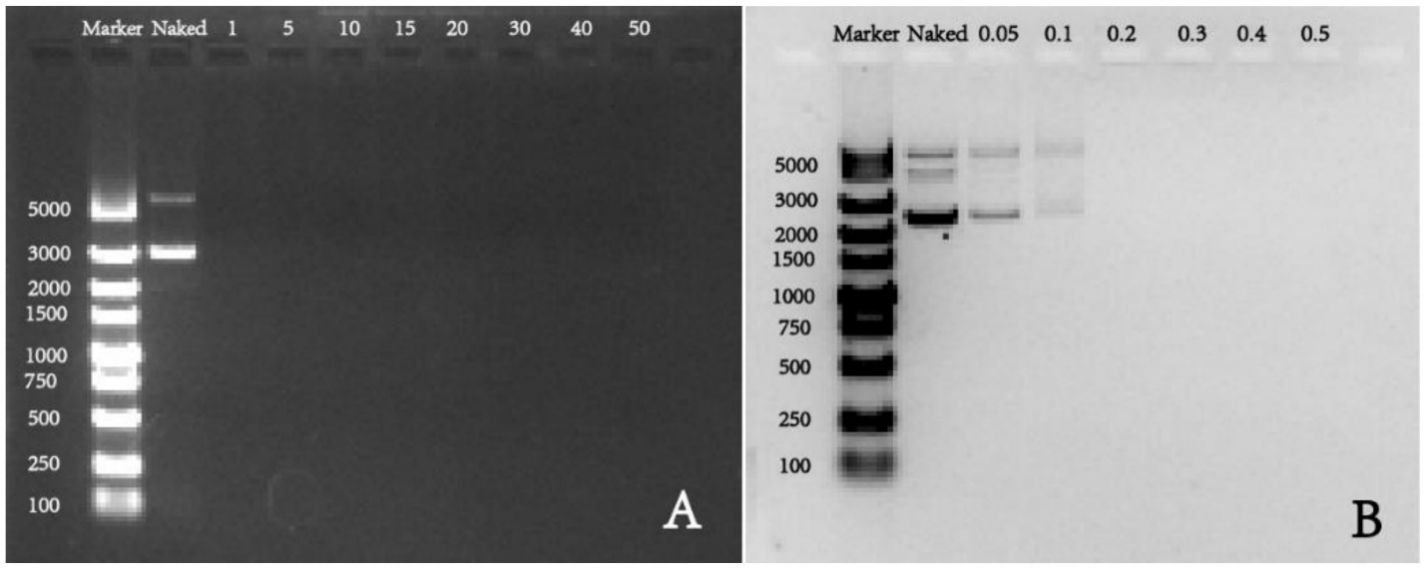
Agarose gel electrophoresis of PDAPEI/pDNA polyplexes at **(A)** 0, 1, 5, 10, 15, 20, 30, 40, and 50 w/w ratios; **(B)** 0, 0.05, 0.1, 0.2, 0.3, 0.4, and 0.5 w/w ratios.

For gene vectors, the binding ability and condensation ability are of great significance to protect pDNA from degradation, and they are evaluated by agarose gel electrophoresis in this study. As shown in the Figure 2A, we found that all groups with w/w ratios of 1, 5, 10, 15, 20, 30, 40, and 50 present had no band in the gel, which means at all these ratios, pDNA mobility was in a complete retardation. The retarded mobility indicated that the synthetic polymer PDAPEI could warp and condense pDNA into polyplexes effectively in these cases. To get a clear understanding in the condensation ability of this polycationic material PDAPEI, low w/w ratios (0.05, 0.1, 0.2, 0.3, 0.4, and 0.5) were performed (Figure 2B). As is illustrated, with the increase of w/w ratio, DNA band became weaker, demonstrating that the addition of PDAPEI into the pDNA solution could lead to a correspondingly smaller pDNA leakage. When w/w ratio was at 0.1, pDNA in solution was almost condensed by PDAPEI completely and the prepared polyplex showed weak leakage (Figure 2B); thus, the effective condensation and protection were ensured at higher w/w ratios.

#### Size, zeta potential, and morphology measurements

**Figure 3:**
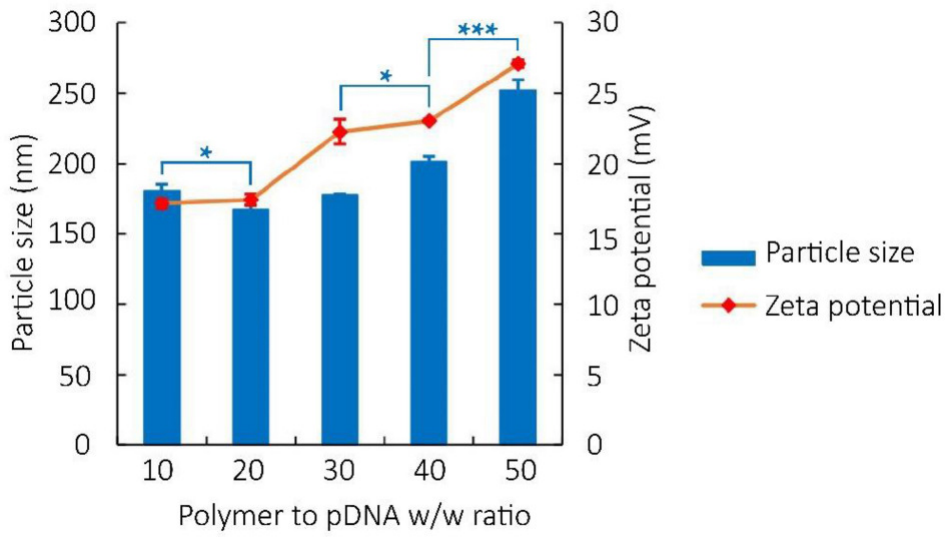
Particle size and Zeta potential of PDAPEI/pDNA polyplexes at 10, 20, 30, 40, and 50 w/w ratios in water. ^*^p < 0.05, ^***^P < 0.001; *n* = 3 per group; data are means ± SD.

Particle size, zeta potential, and surface physicochemical property of polyplex not only reflect indirectly the binding and condensation ability of vectors but also play important roles in the process of cell endocytosis and gene release (van der Aa et al., 2007; Xiang et al., 2012b). In our study, results of particle size measurement of PDAPEI/pDNA polyplex at various w/w ratios indicated an average size ranging from 160 to 250 nm (Figure 3). As can be seen, after a small decrease at the 20 w/w ratio, the particle sizes of polyplex showed a positive correlation with the w/w ratio.

**Figure 4:**
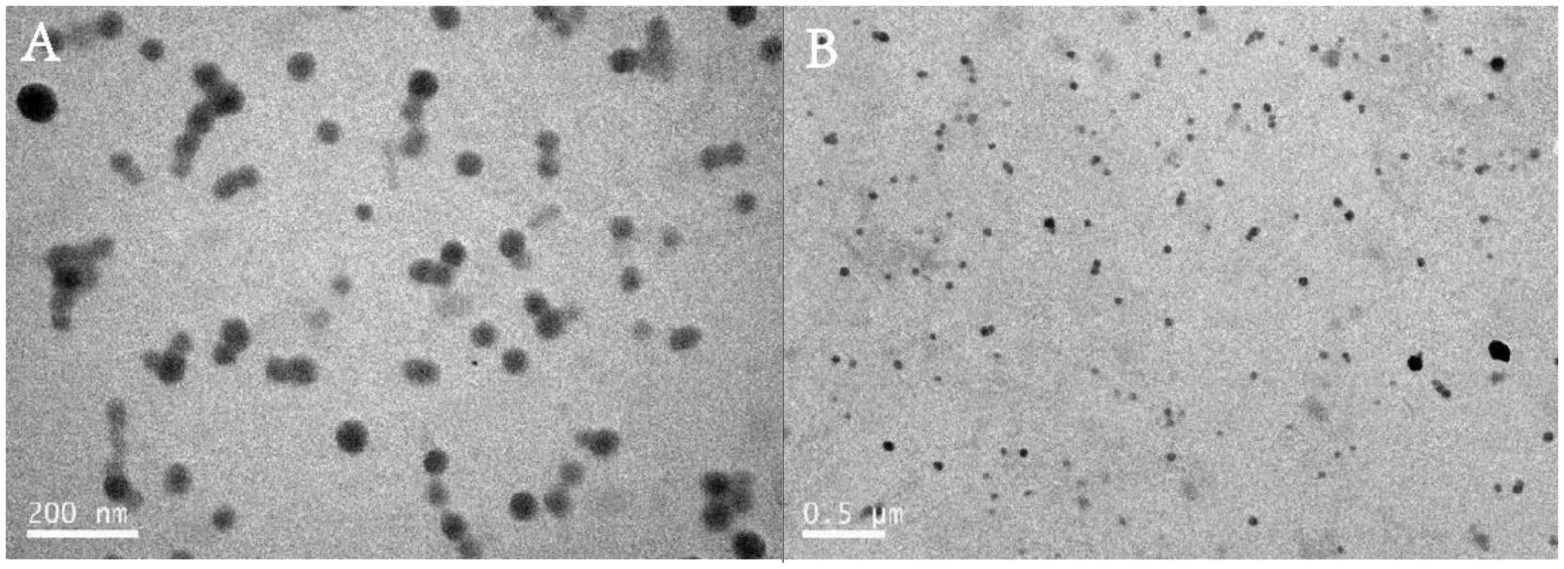
Transmission Electron Microscope (TEM) images of PDA/pDNA polyplexes at 40 w/w ratio with the scale of **(A)** 200 nm; **(B)** 0.5 μm.

The zeta potential of polyplex was stabilized between 17 and 27 mV, implying the ability to physically attach to the negatively charged cell surface (Kabanov and Kabanov, 1995; Figure 3). TEM images also confirmed that polymer PDAPEI at 40 w/w ratio could condense pDNA into spherical nanoparticles with uniform size and morphology (Figure 4), consistent with the result in particle size measurement.

**Figure 5:**
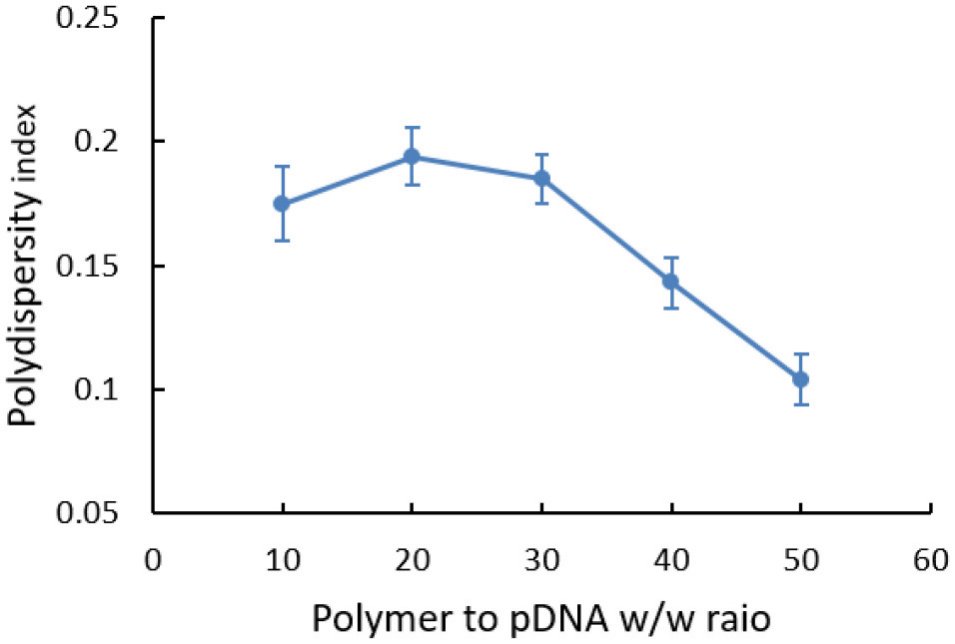
Polydispersity of PDA/pDNA polyplexes at 10, 20, 30, 40, and 50 w/w ratios in water.

PDI of polyplex at different w/w ratios was also analyzed (Figure 5). Generally, the dispersity index was not high (all < 0.30). However, we could find that polymers were in broad polydispersity when it was at the w/w ratio of 10, 20, and 30, illustrating relatively unstable systems that nanoparticles probably had mild aggregate in this state. While for polyplexes with higher w/w ratios, PDI was stabilized between 0.10 and 0.15, which indicated a narrow dispersity of polyplex and implied the possibility for cell endocytosis in theory.

### *In vitro* cell transfection

**Figure 6:**
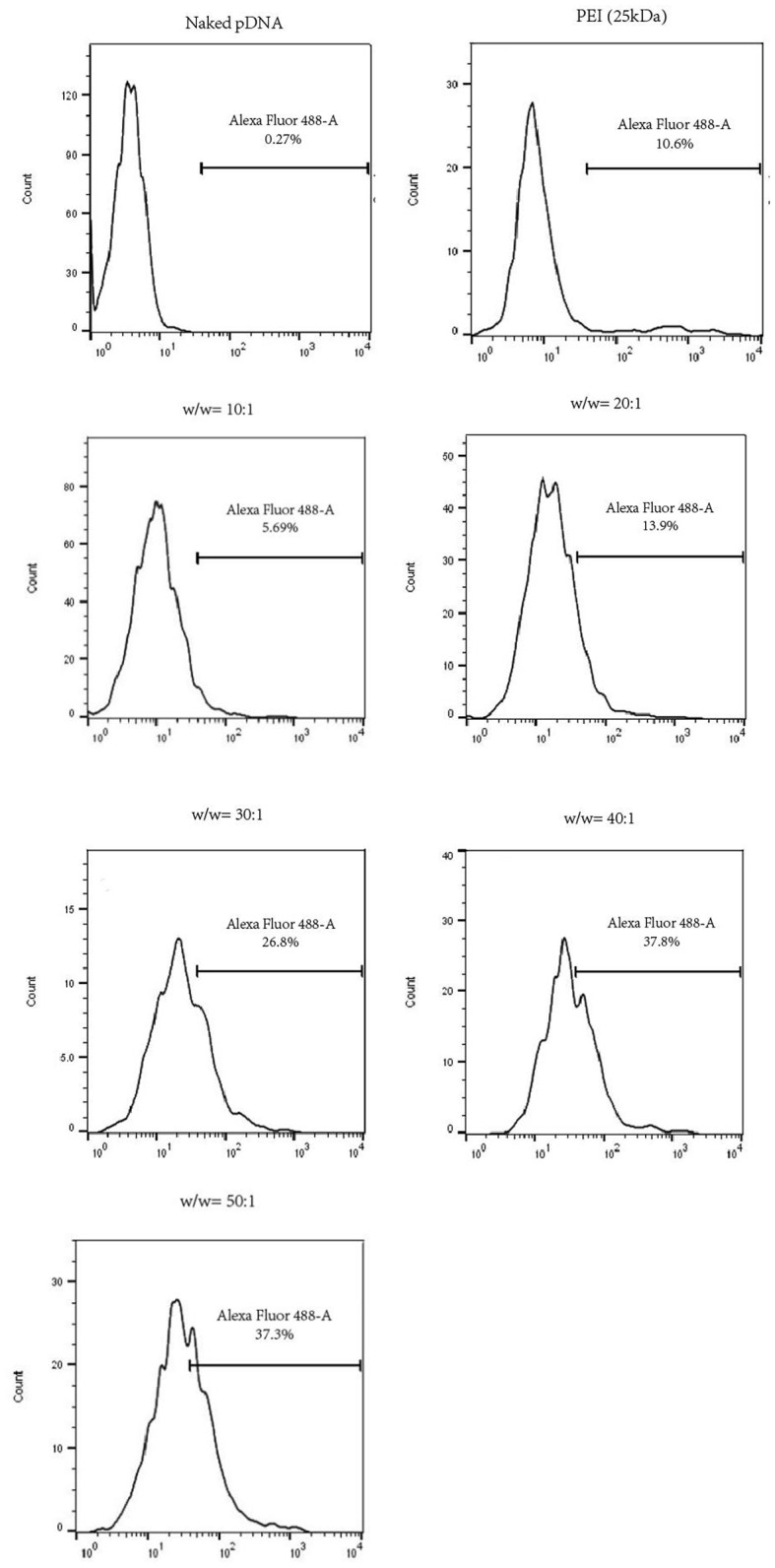
Flow cytometry analysis of naked pDNA, PEI (25k Da)/pDNA, PDAPEI/pDNA polyplexes at 10, 20, 30, 40, and 50 w/w ratios. Transfection efficiency in HUVECs was quantified by the flow cytometer in the form of GFP positive cells.

In the study, HUVECs successfully transfected by pGFP could emit green fluorescence at the wavelength of 488 nm. To identify the transfection efficiency of polyplexes at various w/w ratios, we utilized flow cytometer to analyze the results quantitatively. As is illustrated in Figure 6, cells in the naked pDNA group almost expressed no GFP (0.27%), whereas transfection efficiency of HUVECs treated with PDAPEI/pDNA polyplex showed a positive correlation with the w/w ratio. PEI (25k Da)/pDNA had a moderate transfection efficiency (10.6%), ranking between 10:1 and 20:1 w/w ratios of PDAPEI/pDNA polyplex (5.69–13.9%). A significant increase in transfection efficiency could be seen in PDAPEI/pDNA groups from 20:1 to 50:1 w/w ratios. However, high transfection efficiency should not be seen as the only determinant factor in our selection of the optimal carrier to pDNA ratio, since the cell viability matters as well (Xiang et al., 2012b). The cell cytotoxicity of polyplex is analyzed in the following experiment.

### *In vitro* cytotoxicity

**Figure 7:**
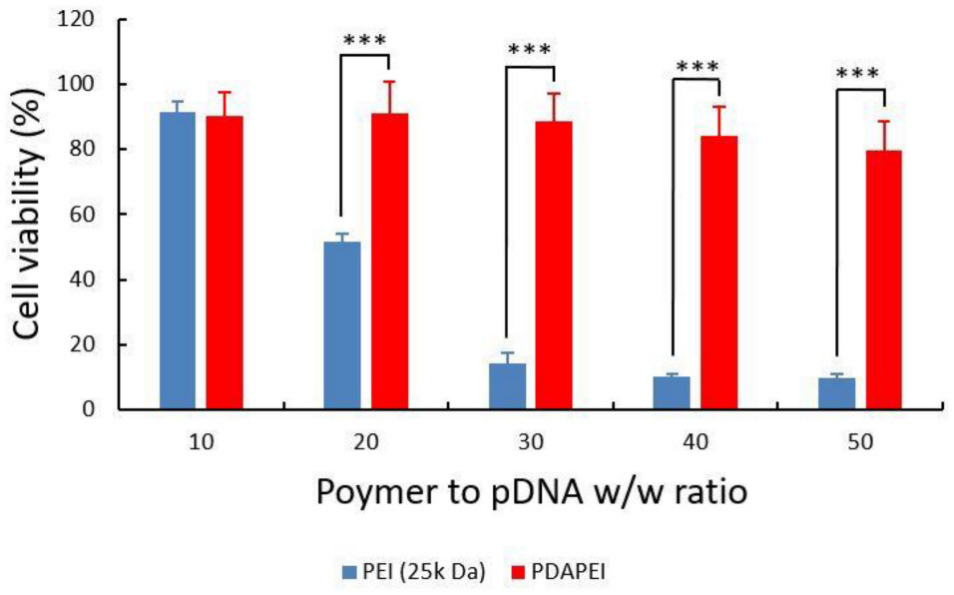
Viability of HUVECs transfected by PEI (25k Da)/pDNA polyplex, PDAPEI/pDNA at 10, 20, 30, 40, and 50 w/w ratios. ^***^P < 0.001; data are means ± SD.

HUVECs viability was calculated and shown in Figure 7. In general, the cytotoxicity of PEI (25k Da)/pDNA polyplex was higher than that of PDAPEI/pDNA polyplex. With the increase of w/w ratio, cell viability of PEI (25k Da)/pDNA polyplex decreased dramatically. In a sharp contrast, the viability of cells treated with PDAPEI/pDNA polyplex remained relatively stable at different w/w ratios. There exists a significant difference in cell viability between PEI (25k Da)/pDNA polyplex control group and PDAPEI/pDNA polyplex group at 20, 30, 40, and 50 w/w ratios (^***^P < 0.001).

### *In vivo* expression of VEGF-A protein

**Figure 8:**
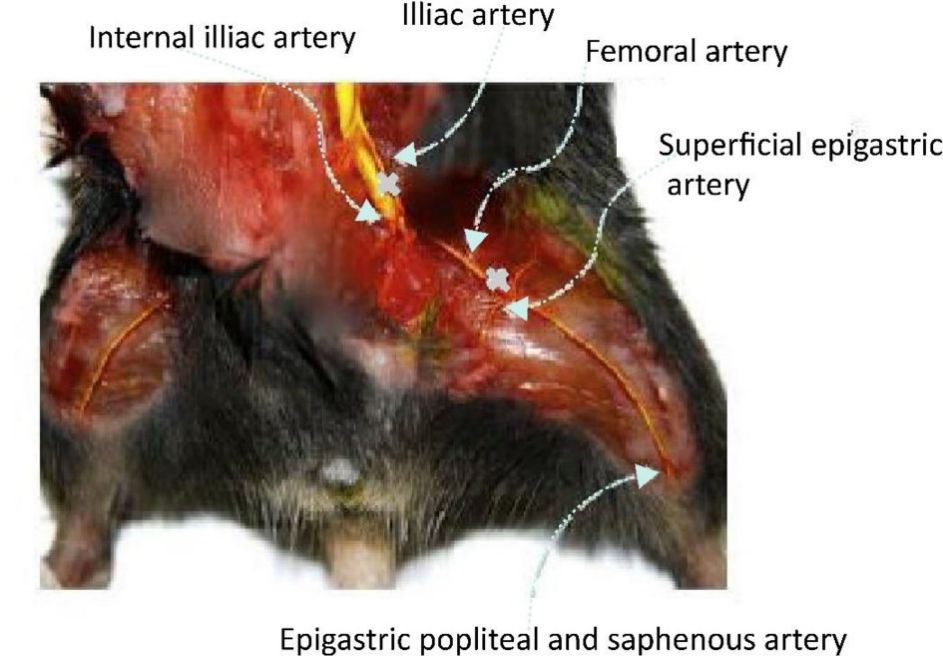
Schematic illustration of hind limb ischemia model. Ligations were made in the femoral artery at the proximal and distal sites.

**Figure 9:**
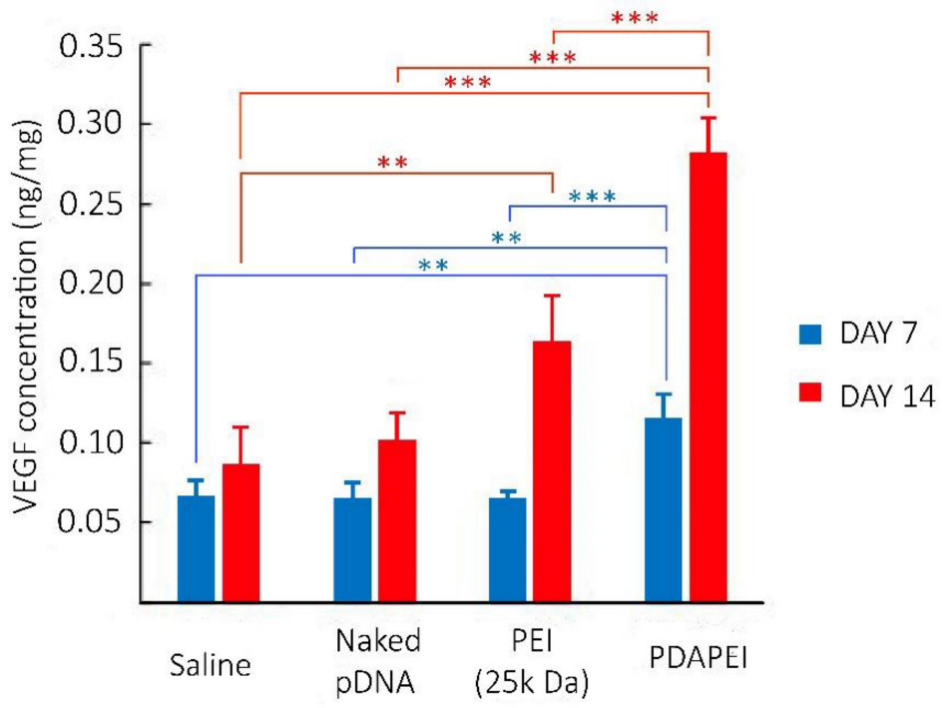
Expression level of VEGF-A protein in hind limb homogenate of saline, naked pDNA, PEI (25k Da)/pDNA and PDAPEI/pDNA groups at Day 7 and Day 14. ^**^P < 0.01, ^***^P < 0.001; *n* = 5 per group; data are means ± SD.

The schematic illustration of hind ischemia model in this study is shown in Figure 8. To assess the transfection efficiency of polyplexes and evaluate the efficacy of treatment in C57/BL6J mice, we detected the expression level of VEGF-A protein in hind limb homogenate (Figure 9). From the figure, we could find that for most groups, the average expression level of VEGF-A at Day 14 was higher than that at Day 7. In addition, compared with all other groups, our PDAPEI group had a significantly higher amount of VEGF-A expression both at Day 7 and Day 14 (^**^P < 0.01, ^***^P < 0.001).

### Immuno-histological examination and microvessel quantification

**Figure 10:**
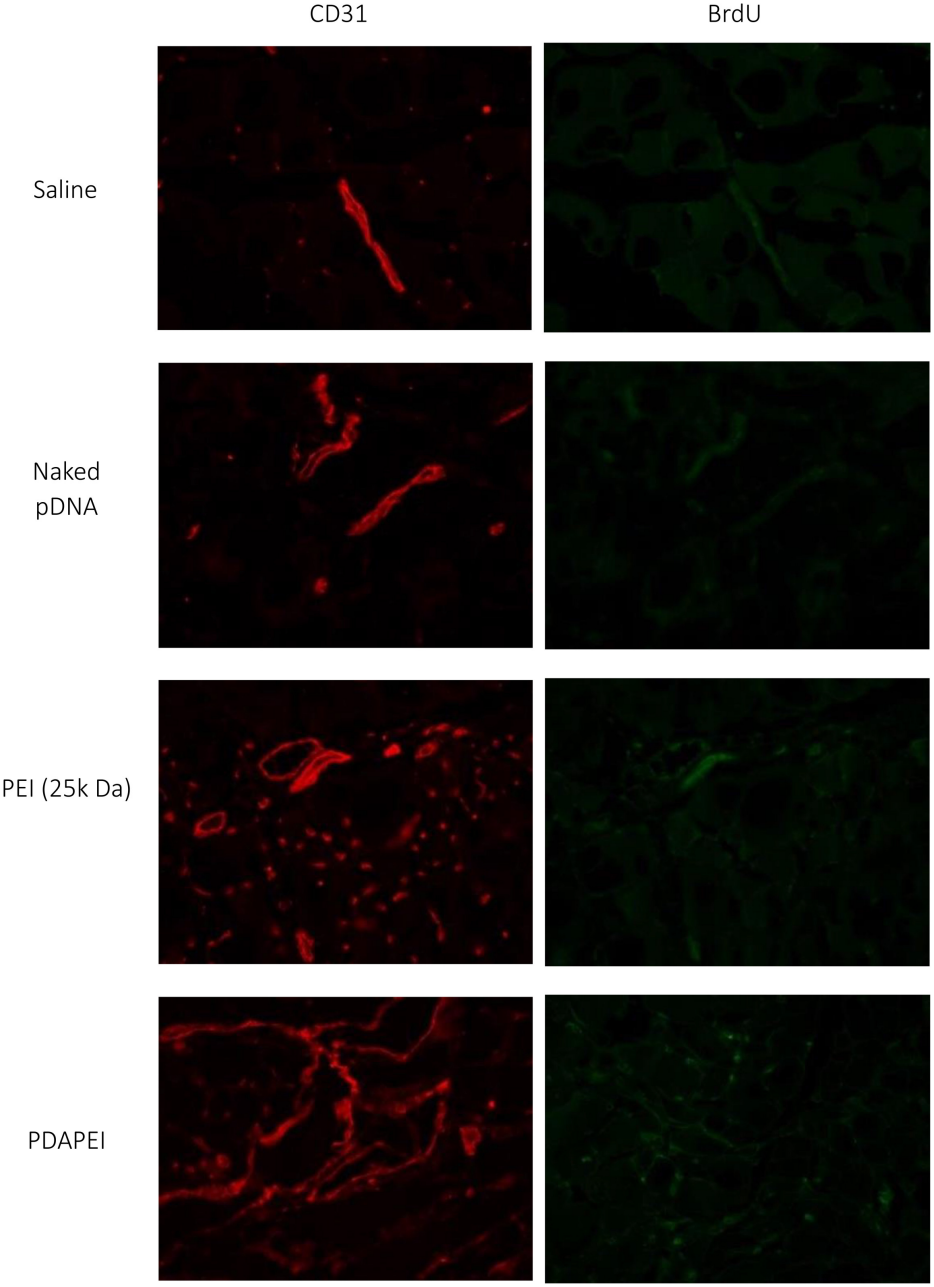
Representative images of double immunofluorescent staining for CD31 and BrdU of ischemic hind limbs in groups of saline, naked pDNA, PEI (25k Da)/pDNA and PDAPEI/pDNA groups at Day 28.

**Figure 11:**
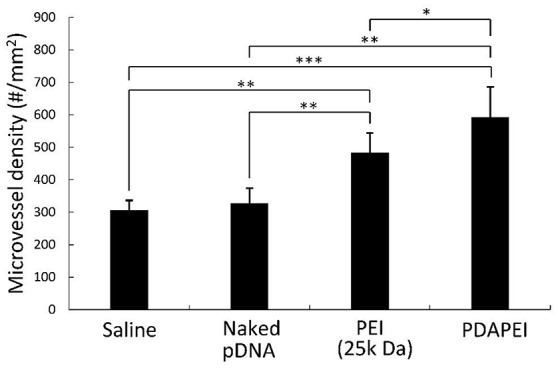
Microvessel densities in peripheral muscle tissues of C57/BL6J mice in groups of saline, naked pDNA, PEI (25k Da)/pDNA and PDAPEI/pDNA groups at Day 28. ^*^p < 0.05, ^**^P < 0.01, ^***^P < 0.001; *n* = 5 per group; data are means ± SD.

Figure 10 demonstrates the examination of peripheral hind limb muscle tissues after double immunofluorescent staining for CD31 and BrdU. As can be seen, microvessels in saline group and naked pDNA group were sparse, whereas densities PDAPEI and PEI (25k Da) groups were higher. The microvessel densities were quantified as shown in Figure 11. Muscle tissue microvessel densities were significantly greater both in the PEI (25k Da) (483.96 #/mm^2^) and PDAPEI (593.11 #/mm^2^) groups compared to the saline (blank; 305.81 #/mm^2^) and naked pDNA (negative; 327.49 #/mm^2^) groups (^**^P < 0.01, ^***^P < 0.001). In addition, there were significantly more microvessels in PDAPEI group than the 25k Da PEI (positive) group (^*^P < 0.05).

## Discussion

CLI constitutes a life-limiting and life-threatening disease, with poor prognosis and serious mortality (Kitrou et al., 2017). In this case, VEGF-A protein is considered to be the prominent growth factor in therapeutic angiogenesis (Bobek et al., 2006; Ganta et al., 2017). Consequently, a clinical therapy targeting VEGF-A protein would be particularly efficacious in the treatment of CLI.

VEGF protein and VEGF pDNA have been the attractive alternatives in the CLI preclinical treatment (Bauters et al., 1995; Laitinen et al., 2000; Manninen and Makinen, 2002). We have reported that our pre-synthetic polymer PDAPEI is an effective non-viral vector in delivering siRNA for gene silencing (Che et al., 2016; Song et al., 2016). In this study, we demonstrated the more effectiveness of the PDAPEI as a VEGF-A pDNA delivery vehicle in the CLI treatment than PEI (25KD).

The PDAPEI/pDNA polyplexes at different ratios had an average size ranging from 160 to 250 nm (Figure 2). With more polymer PDAPEI added in the formula, the increase in particle size after 20 w/w ratio was actually contrary to our expectation. The condensation ability of pH-sensitive polycationic materials depends on the positive charge density (Yue and Wu, 2013), as a result of which we believe that a higher w/w ratio of polyplex should correspond to a smaller particle size. The surprising result was analyzed for two reasons. For one thing, with an increasing w/w ratio of polyplex, although the amino groups in PDAPEI increase in number which could be pronated to generate positive charge, what we could not neglect is that the pH of the solution also rises. This rise in pH affects the pronation of amino groups in PDAPEI in turn and reduces the positive charge density. When the decrease of positive charge density outweighs the increase of amino pronation density, the addition of PDAPEI would weaken the capacity of condensing DNA. For another thing, a higher w/w ratio means a larger occupation in volume, which is also an ignorable factor in this microscale experiment.

There exists a significant difference in cell cytotoxicity between PDAPEI/pDNA polyplex and PEI (25k Da)/pDNA control group at 20, 30, 40, and 50 w/w ratios (Figure 7). High molecular weight PEI is often expected to have high transfection efficiency, whereas the cytotoxicity is high as well due to the high cationic charge density or the difficulty of biodegradability *in vivo* (Fischer et al., 1999; Morimoto et al., 2003; Moghimi et al., 2005; Wei et al., 2015). The polycationic material PDAPEI is synthesized by two basic units, PEI with small molecular weight and PDA, both of which are non- or low-toxic. The design of PDA as a building block in drug discovery has been tried in several reports (Katayev et al., 2007, 2008; Che et al., 2016; Song et al., 2016). The formed bisimine bond through the conjugation of dialdehyde and amine is believed to be destroyed in the acid environment (Kim et al., 2005). When the PDAPEI/pDNA polyplex was delivered into endosomes, it could rupture the endosome through the hypothesized “proton sponge effect” and be metabolized to release gene in this low acidic medium. As a result, PDAPEI/pDNA polyplex had a better performance on *in vitro* cytotoxicity than that of PEI (25k Da)/pDNA polyplex control group.

Combining all experimental results in physiochemical properties, cell transfection efficiency and cytotoxicity of PDAPEI/pDNA polyplex, we chose 40:1 (w/w) ratio as our optimum. The optimal PDAPEI/pDNA polyplex showed a significantly higher level in VEGF-A expression compared with all other groups both at Day 7 and Day 14 (Figure 9). In addition, *in vivo* immuno-histological examination clearly showed that the microvessel density in PDAPEI/pDNA-treated mice was significantly more than that in control groups, which successfully confirmed the efficacy of our VEGF-A pDNA delivery using biodegradable polycationic material PDAPEI. In this study, the mice treated with PDAPEI/pDNA polyplex at 40 w/w ratio had an increased local tissue concentration of VEGF-A protein and increased microvessel density.

Indeed, it is very exciting to look into the future about the potential application of our PDAPEI/pVEGF-A polyplex in human PAD. Currently there is still no clinically effective drug therapy for PAD and the amputation rates of this disease have not significantly changed during the past 30 years (Tongers et al., 2008). Modern therapies with higher efficiency and safety such as, the angiogenic gene therapy in the present study are urgently needed. The beneficial outcome of VEGF-A gene delivery with our synthetic polymer PDAPEI in animal models makes it reasonable for us to expect that it could be a substantially potent therapy in clinical treatments of human ischemic diseases. However, much systematic work on the optimal dose, method of administration, etc., is still needed to be done before clinical studies. Apart from the bright future of VEGF-A gene therapy shown in this study, another aspect we cannot neglect is the immense potential of our synthetic biodegradable polymer PDAPEI. With low or no cell cytotoxicity (Figure 7), PDAPEI might be a feasible biomaterial for human use especially when at a small dose. Thus, we are positive to expect that this novel polymer could be used more widely in the biomedical field in the future.

Despite the impressive results shown here, there are still some aspects that can be further developed in this study. We measured the expression level of VEGF-A protein in hind limb homogenate and found expressed protein in PDAPEI group was significantly more both at Day 7 and Day 14. However, there seemed to be a big jump in the expression level from Day 7 to Day 14 in this group. What we expected was that the increase of expression level of VEGF-A protein could be correlated with the time, whereas in Figure 10, it was shown that the increase of expression level at Day 7 was much less than half of that at Day 14. Similar phenomenon was also reported in the previous gene delivery studies and it was described as the “lag time” for gene expression (McCarty et al., 2001; Boden et al., 2016). One strategy to resist this potential lag effect is pre-injection, which could confer tissue and rapid onset of necrosis in an early way (Boden et al., 2016). Other methods that could speed up the gene delivery and expression still need to be further explored. Another limitation of the present study was the need of frequent injection to local ischemic tissue, although the restoration of ischemia turned out to be promising. We are now considering a novel strategy to encapsulate the PDAPEI/pDNA polyplex described here to realize the long-term and controlled release of gene, which is expected to enable to reduce the frequency of injection. Furthermore, modified carriers with the ability of specific targeting might also be another strategy to further improve the efficiency and safety of gene therapy.

## Conclusion

In general, combining the experiment results above, we confirmed the binding and condensation ability of our synthetic polycationic material PDAPEI and utilized this biologically responsive vector to deliver VEGF-A pDNA in the murine model of PAD. We could safely draw a conclusion that the novel PDAPEI/pDNA polyplexes at 40 w/w ratio in our study showed a better performance in gene delivery than PEI (25k Da)/pDNA polyplexes, with similar therapeutic effect but lower cytotoxicity. This non-viral carrier delivering VEGF-A pDNA could be a promising strategy for the clinical treatment of CLI.

## Author contributions

GL, ZF, MY, WL, YY, YO, MJ, and WY participated in its design, searched databases, extracted and assessed studies, helped to draft the manuscript. WY conceived the initial idea and the conceptualization, participated in the data extraction and analysis, revised the manuscript, YO, MJ, GL, WL, and MY also participated in revising the manuscript. All authors read and approved the final manuscript.

### Conflict of interest statement

The authors declare that the research was conducted in the absence of any commercial or financial relationships that could be construed as a potential conflict of interest. The reviewer DH and handling Editor declared their shared affiliation, and the handling Editor states that the process met the standards of a fair and objective review.
